# Non‐smoking and Non‐drinking Oral Cancer Patients Are at Higher Risk of Second Primary Tumours

**DOI:** 10.1111/odi.15235

**Published:** 2024-12-30

**Authors:** Pepijn J. P. van der Aa, Max J. H. Witjes, Bert van der Vegt, Ed Schuuring, Koos Boeve, Grigory Sidorenkov, Geertruida H. de Bock, Sebastiaan A. H. J. de Visscher

**Affiliations:** ^1^ Department of Oral and Maxillofacial Surgery, University Medical Center Groningen University of Groningen Groningen The Netherlands; ^2^ Department of Pathology and Medical Biology, University Medical Center Groningen University of Groningen Groningen The Netherlands; ^3^ Department of Epidemiology, University Medical Center Groningen University of Groningen Groningen The Netherlands

**Keywords:** alcohol, clinicopathological features, oral cancer, recurrence, second primary tumour, smoking

## Abstract

**Objectives:**

This study aimed to explore differences in demographics, tumour characteristics and outcomes in oral squamous cell carcinoma (OSCC) patients with a history of non‐smoking, non‐drinking (NSND) versus smoking and/or drinking (SD).

**Materials and Methods:**

Newly diagnosed OSCC patients undergoing curative surgical treatment were prospectively included in OncoLifeS, a data biobank. Cox regression analysis was performed yielding hazard ratios (HRs) and 95% confidence intervals (95%CIs).

**Results:**

185 patients were included, and 32.4% of patients were NSND; this group represented an older (69 vs. 64.4 years, *p* < 0.01) and more female‐dominated (66.7% vs. 44.5%, *p* = 0.02) population. NSND patients had more tongue tumours (68.3% vs. 46.4%, *p* < 0.01) and few floor‐of‐mouth tumours (1.7% vs. 20.0%, *p* < 0.01). Locoregional recurrence, overall survival and disease‐specific survival risk were similar between the NSND and SD patients. NSND patients had a higher second primary tumour risk compared to SD patients in the multivariable analysis (adjusted HR 3.92, 1.23–12.48, *p* = 0.02).

**Conclusion:**

NSND patients with OSCC have a distinct clinicopathological profile compared to SD patients, with a higher risk of second primary tumours after treatment. These differences in risk profiles should be considered in future OSCC management strategies.

## 1 Introduction

Oral squamous cell carcinoma (OSCC) presents a major health burden worldwide due to anatomical and functional morbidity associated with treatment. In the Netherlands, oral cancers have a reported incidence of 4.2 per 100,000 in males and 3.3 per 100,000 in females. Unfortunately, in the last three decades, the incidence rates increased by 0.7% for males and 1.8% for females per year in the Netherlands (Braakhuis, Leemans, and Visser 2014; van Dijk et al. 2016; IKNL, 2024). Important etiological factors of OSCC are tobacco use and alcohol consumption, in which combined consumption increases the risk of occurrence even further (Maasland et al. 2014; Mello et al. 2019).

In the past decades, much attention has been given to stimulate the public to stop or not start smoking worldwide. Despite the decline in tobacco use from 38.0% to 20.2% between 1990 and 2021 in the Netherlands, there was an increase in OSCC cases from 507 to 913 in the same period (Bruggink 1991; van Laar et al. 2021; NCR 2024). It is not fully understood what drives this increase in incidence. It is known that certain potential malignant disorders or conditions may yield OSCC through unknown mechanisms, for example, leukoplakia and lichen planus (Warnakulasuriya et al. 2021). For a small portion of the patients, the aetiology of their OSCC will not be known. There is debate about whether non‐smoking and non‐drinking (NSND) patients should be regarded as a separate subgroup of OSCC, due to lack of classic etiological factors, as was suggested in a recent review (Adeoye et al. 2021). This review reveals that NSND patients tend to be older, predominantly female and frequently present with tumours located on the oral tongue. Some studies showed a lower survival rate for NSND female patients, but in most studies disease‐specific prognosis and treatment response were comparable between NSND and smoking and/or drinking (SD) patients.

Currently, there is no consensus on NSND OSCC patient prognosis. Most of the published studies rely on retrospective data and are prone to bias, like missing data, inclusion bias or inclusion of multiple head and neck cancer locations with different aetiologies (Adeoye et al. 2021; Farshadpour et al. 2007). Moreover, detailed knowledge concerning the number and clinical outcomes of NSND OSCC patients in the Netherlands is currently inadequate.

The goal is to study patient characteristics, histopathological characteristics and recurrence rates in OSCC patients with a history of smoking and/or drinking (SD) versus NSND patients. For this purpose, data from a well‐defined, prospectively included patient cohort gathered by using a broad range of validated instruments for the assessment of clinical, socio‐demographic and behavioural factors (the OncoLifeS data‐biobank) is used.

## 2 Materials and Methods

### 2.1 Study Design

This study is a single‐centre prospective observational cohort study, using data from a hospital‐based data‐biobank for oncology patients, named OncoLifeS (Oncological Life Study) (Sidorenkov et al. 2019). The OncoLifeS initiative can be used to evaluate and improve treatment for patients who may otherwise never be included in clinical trials, providing additional information across a broader spectrum of conditions, using multiple questionnaires described in detail before. The OncoLifeS data‐biobank has been established in 2014 at the University Medical Center Groningen (UMCG). After written consent, all patients with a diagnosis of cancer are included. OncoLifeS has been approved by the local Medical Ethical Committee (approval number 2010/109). The present study protocol (Reference number: 202100015) was approved by the OncoLifeS scientific board.

### 2.2 Study Population

Patients were included for the period June 2014 to July 2022. Inclusion criteria were (a) validated participant of OncoLifeS, with written informed consent, (b) pathological diagnosis of squamous cell carcinoma, (c) primary tumour located in the oral cavity subsites (ICD‐O‐3 C02–C06), (d) primary surgical treatment with curative intent, and (e) patient older than 18 years of age. Exclusion criteria were (1) no baseline questionnaires completed by patient, this included smoking and drinking behaviour; and (2) patients with a head or neck tumour prior to OncoLifeS participation. All patients were discussed in a multidisciplinary tumour board and treated following the Dutch Workgroup Head–Neck tumour guidelines (NWHHT 2004).

### 2.3 Data Collection

Alcohol and tobacco use were assessed using patient questionnaires. Tobacco usage was classified as smoking behaviour, divided into current smokers and non‐smokers. Smokers were defined as patients smoking at the time of diagnosis or who had a history of smoking. Non‐smokers were defined as patients smoking less than 100 cigarettes in a lifetime. To assess the dosage of tobacco use, cigarettes smoked per day and number of years smoking were obtained, calculating pack years (PY) smoked (IARC 2004). To assess the dosage of alcohol consumption, the number of drinks (units of alcohol) per week was calculated. The number of alcohol consumptions per day and number of days drinking per week were obtained. Alcohol consumption was classified as drinking behaviour, divided into drinkers and non‐drinkers. Drinkers were defined as harmful drinking at time of diagnosis (> 14 units of alcohol per week for men, > 7 units of alcohol per week for women) or as patients that have a history of harmful drinking alcohol (IARC 2010). To define combined intoxication behaviour, groups were divided into drinkers and/or smokers (SD) and non‐drinkers plus non‐smokers (NSND).

Baseline covariates included age at diagnosis, patient sex, Body Mass Index (BMI), Groningen Frailty Indicator (GFI) (Schuurmans et al. 2004), age‐adjusted Charlson Comorbidity Index (CCI) (Charlson et al. 1994) and history of previous other malignancies. Tumour characteristics were assessed using the AJCC/UICC TNM classification according to the 8th edition from 2018 to 2022 (Amin, Edge, and Greene 2017). Because of the changes in TNM classification from the 7th to 8th edition during the inclusion period, the raw histopathological data were used in this study: tumour size in millimetres, depth of invasion in millimetres, lymph node metastasis, extranodal extension, lymphovascular invasion and perineural invasion. Resection margins ≥ 5 mm were defined as clear. If a reresection was performed, resection margin status after reresection was used for analyses. Neck dissection, sentinel node biopsy, radiotherapy and chemotherapy data were also collected.

### 2.4 Outcome Measures

During follow‐up, the following second events were registered: locoregional recurrence, distant metastasis and second primary tumours (SPTs). Recurrence was defined as local and/or regional recurrence. Local recurrence (LR) was described as a pathological proven diagnosis in the same ICD‐0‐3 topography code, side and same histological subtype as the index tumour, occurring at a minimum of 6 weeks after treatment and within 2 years of the initial diagnosis. Regional recurrence (RR) was defined as recurrent tumour occurring within the lymph neck nodes at a minimum 6 weeks after treatment and within 2 years of initial diagnosis (Chegini et al. 2021). Distant metastasis (DM) was defined as an OSCC that has spread to other organ systems after treatment based on imaging patterns often confirmed by histology. Time to recurrence was calculated from the date of pathological diagnosis of the index tumour until the date of pathological diagnosis of recurrence in months. SPTs were defined as head and neck cancer, arising in a different localisation (at least 2 cm away) or in the same location after > 2 years of index tumour (Braakhuis et al. 2002). Secondary outcomes were overall survival (OS, death from any cause) and disease‐specific survival (DSS, death due to (metastasis of) index tumour). OS was calculated from the date of pathological diagnosis of the index tumour until the date of death, independent of the cause of death, in months. DSS was calculated from the date of pathological diagnosis of the index tumour until the date of death, related to OSCC, in months. Follow‐up ended at either the date of death, data‐censoring date December 8, 2022, or date of lost to follow‐up (e.g. in case of emigration).

### 2.5 Statistical Analysis

Statistical analysis was performed using IBM SPSS Statistics for Windows (Version 25. Armonk, NY: IBM Corp). All baseline categorical data were presented as numbers (*N*) and their percentages (%). Continuous data were presented as mean with standard deviation (SD). Descriptive statistics were summarised as frequencies, percentages and median ± IQR. Continuous data were tested using the Student's *t*‐test or the Mann–Whitney *U* test for normally or skewed distributed data, respectively. Chi‐squared test was used to test for differences between categorical data. If samples had an observed count of < 5, Fisher's exact test was used. The Kaplan–Meier method was used to derive estimates for the percentage of second events, OS and DSS at 1, 2 and 5 years. To evaluate the impact of the baseline characteristics on the outcome, survival analyses were performed. Univariate Cox proportional hazard models were applied to identify significant factors of second events, OS and DSS for including into the multivariate model. To adjust for the effects of potential confounders, multivariable Cox proportional hazard models were performed for the adjusted hazard ratio. A stepwise backward selection of univariate significant variables (*p* < 0.05) was used until only significant variables, smoking/drinking status, age and sex remained. Multivariable Fine‐Grey's competing risk model was utilised for sensitivity analysis to assess whether death/other events were competing events [Fine and Gray 1999]. R software (version 4.4.1) using the cmprsk package was used. We only included patients with primary surgical treatment with curative intent. As this may limit the generalisability of the results, we also analysed potential differences between included and non‐included patients based on treatment exclusion. All tests were two‐sided, (adjusted) hazard ratios (HRs), confidence intervals of 95% (95%CIs) were used and *p*‐values < 0.05 were considered statistically significant.

## 3 Results

### 3.1 Demographics

Between 2014 and 2022, 185 patients met the eligibility criteria and were included in this study. A flowchart of patient inclusion is added to the (Figure S1). To compare baseline characteristics, all parameters were analysed and stratified by the NSND and SD groups (Table 1). Sixty patients (32.4%) were NSND, and this group was significantly older than SD patients (68.8 vs. 64.4 years, *p* < 0.01). Gender distribution was dominated by women in the NSND group (66.7% vs. 44.5%, *p* = 0.02). Other patient characteristics, such as BMI, comorbidity, frailty and history of other cancers, did not significantly differ between the two groups. The NSND group had mainly tongue tumours and less floor‐of‐mouth tumours compared to the SD group, 68.3% versus 46.4% and 1.7% versus 20.0%, respectively (*p* < 0.01). Staging was not different as 60% of patients in the NSND group had advanced stage (TNM III and IV) versus 53.6% in the SD group (*p* = 0.41). Margin status after surgery was similar between groups (14.4% vs. 8.3%, *p* = 0.12). No differences were found for the number of sentinel node biopsies (*p* = 0.40), neck dissections (*p* = 0.72) or adjuvant treatment which consisted of reresection or (chemo)radiation (*p* = 0.70).

**TABLE 1:** Patient, tumour and treatment characteristics stratified by NSND and SD groups, (*N* %), unless specified otherwise (*N* = 185).

Variables	Total		NSND		SD		*p*
Total	185	100%	60	32.4%	125	67.6%	
Age							
Years (mean ± SD)	65.9 ± 11.5		69.0 ± 12.1		64.4 ± 10.9		**< 0.01**
Gender							
Female	101	54.8%	40	66.7%	61	44.5%	**0.02**
Male	84	45.2%	20	33.3%	64	55.5%	
BMI							
< 18.5 kg/m^2^	8	4.3%	0	0%	8	6.4%	0.12
18.5–24.99 kg/m^2^	82	44.3%	24	40.0%	58	46.4%	
25–29.99 kg/m^2^	53	28.6%	21	35.0%	32	25.6%	
≥ 30 kg/m^2^	42	22.7%	15	25.0%	27	21.6%	
CCI							
< 5	81	43.8%	25	41.7%	56	44.8%	0.69
≥ 5	104	56.2%	35	58.3%	69	55.2%	
GFI							
< 4	119	75.8%	39	73.6%	80	76.9%	0.64
≥ 4	38	24.2%	14	26.4%	24	23.1%	
Previous other cancers							
0	158	84.9%	55	82.1%	103	86.6%	0.33
1	23	12.4%	8	13.4%	14	11.8%	
2	1	0.5%	0	0%	1	0.8%	
3 or more	4	2.2%	3	4.5%	1	0.8%	
Tumour site							
Tongue	99	53.5%	41	68.3%	58	46.4%	**< 0.01**
Gum	36	19.5%	10	16.7%	26	20.8%	
Floor of mouth	26	14.1%	1	1.7%	25	20.0%	
Palate	4	2.2%	2	3.3%	2	1.6%	
Buccal mucosa	20	10.8%	6	10.0%	14	11.2%	
Differentiation grade							
Well	50	27.2%	20	33.9%	30	24.0%	0.57
Moderate	114	62.0%	33	55.9%	81	64.8%	
Poor	10	5.4%	3	5.1%	7	5.6%	
Unknown	10	5.4%	3	5.1%	7	5.6%	
Tumour size							
≤ 20 mm	102	55.6%	35	58.3%	67	53.6%	0.82
20–40 mm	61	34%	18	30.0%	43	34.4%	
> 40 mm	22	10.4%	7	11.7%	15	12.0%	
Depth of invasion							
≤ 5 mm	79	42.2%	29	48.3%	50	40.0%	0.80
> 5 and ≤ 10 mm	56	30.1%	16	26.7%	40	32.0%	
> 10 mm and ≤ 20 mm	38	20.5%	12	20.0%	26	20.8%	
> 20 mm	11	5.9%	3	5.0%	8	6.4%	
Unknown	1	0.5%	0		1	0.8%	
Resection margins^a^							
≥ 5 mm	92	49.7%	34	56.7%	58	46.4%	0.12
4–4.99 mm	19	10.3%	3	5.0%	16	12.8%	
3–3.99 mm	20	10.8%	8	13.3%	12	9.6%	
2–2.99 mm	15	8.1%	7	11.7%	8	6.4%	
1–2 mm	16	8.6%	3	5.0%	13	10.4%	
< 1 mm	23	12.4%	5	8.3%	18	14.4%	
Perineural invasion							
Absent	144	77.8%	43	71.7%	01	80.8%	0.16
Present	41	22.2%	17	28.3%	24	19.2%	
LVI							
Absent	164	88.6%	54	90.0%	110	88.0%	0.69
Present	21	11.4%	6	10.0%	15	12.0%	
Pathological N status							
N0	110	58.6%	35	58.3%	75	60.0%	0.83
N+	75	41.4%	25	41.7%	50	40.0%	
Extranodal extension^b^							
Absent	55	73.3%	17	68.0%	38	76.0%	0.46
Present	20	26.7%	8	32.0%	12	24.0%	
8th AJCC pTNM stage							
I	57	30.8%	18	30.0%	39	31.2%	0.43
II	25	13.5%	6	10.0%	19	15.2%	
III	32	17.3%	14	23.3%	18	14.4%	
IV	71	38.4%	22	36.7%	49	39.2%	
Sentinel node biopsy							
None	110	59.5%	37	61.7%	73	58.4%	0.40
Yes	75	40.5%	28	38.3%	52	41.6%	
Neck dissection							
None	55	29.7%	20	33.3%	35	28.0%	0.72
Diagnostic	71	38.4%	21	35.0%	50	40.0%	
Therapeutic	59	31.9%	19	31.7%	40	42.0%	
Adjuvant therapy							
Reresection	24	13.0%	8	13.3%	16	12.8%	0.70
Radiotherapy	67	36.2%	22	36.7%	45	36.0%	
Chemoradiotherapy	12	6.5%	3	5.0%	9	7.2%	
Reresection and RT	6	3.2%	3	5.0%	3	2.4%	
Reresection and chemoradiotherapy	1	0.5%	0	0%	1	0.8%	
None	75	40.5%	24	40.0%	51	40.8%	

Abbreviations: BMI, body mass index kg/m^2^; CCI, age‐adjusted charlson comorbidity index; GFI, groningen frailty indicator; LVI, lymphovascular invasion; RT, radiotherapy. Bold values signify *p*‐value < 0.05.

^a^ If reresection was performed, resection margins after reresection were used.

^b^ Only patients with positive pathological nodal status are counted.

### 3.2 Outcome

**FIGURE 1:**
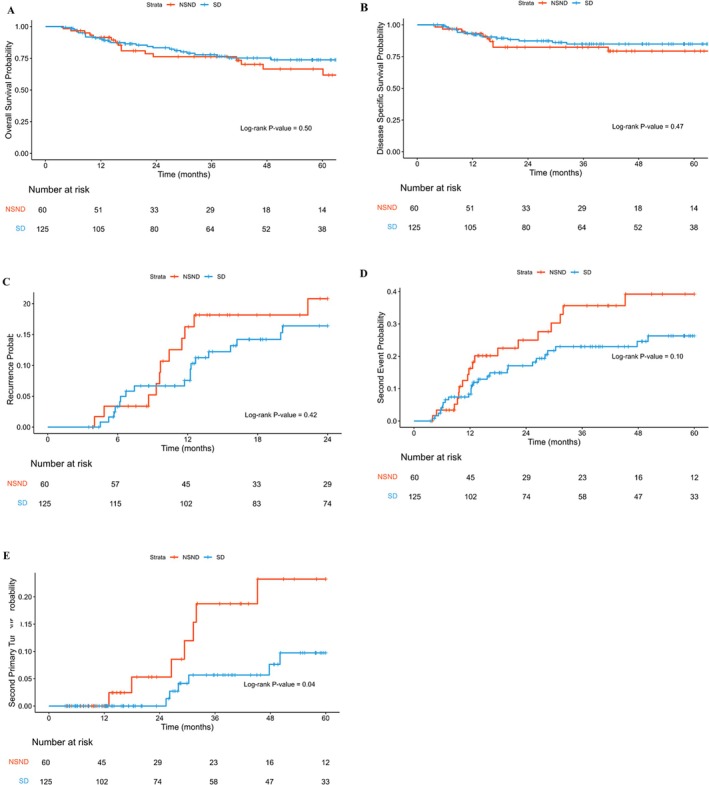
(A) Kaplan–Meier overall survival plot for NSND patients (*N* = 60) compared to SD patients (*N =* 125), log‐rank *p* = 0.50. (B) Kaplan–Meier disease‐specific survival plot for NSND patients (*N* = 60) compared to SD patients (*N =* 125), log‐rank *p* = 0.47. (C) Kaplan–Meier recurrence plot for NSND patients (*N* = 60) compared to SD patients (*N =* 125), log‐rank *p* = 0.42. (D) Kaplan–Meier second event plot for NSND patients (*N* = 60) compared to SD patients (*N =* 125), log‐rank *p* = 0.10. (E) Kaplan–Meier second primary tumour plot for NSND patients (*N* = 60) compared to SD patients (*N =* 125), log‐rank *p* = 0.04.

Median follow‐up time was 36.0 months (interquartile range 14.0–62.5), and no difference in follow‐up time was found between the NSND and SD groups (*p* = 0.30) (Table S1). In total, 24.3% of patients died within 5 years of follow‐up. 14.1% of patients died due to the index tumour and 10.2% of patients died due to other causes. For the total population, the 2‐year OS and DSS were 81.7% and 85.9%, respectively. For the 5‐year OS and DSS, these percentages dropped to 66.6% and 83.2%. When comparing the two groups in Kaplan–Meier analysis for OS, the NSND group showed similar survival estimates compared to the SD group, 61.9% versus 68.6%, respectively (log‐rank, *p* = 0.49) (Figure 1A). In the Kaplan–Meier analysis, DSS was 79.5% for NSND versus 84.9% in the SD group (log‐rank, *p* = 0.47) (Figure 1B). Cox univariate analysis showed comparable hazard ratios for OS (HR (95% CI) 0.81, 0.44–1.49, *p* = 0.49) and DSS (HR (95% CI) 0.74 0.34–1.64, *p* = 0.46) (Table S2). Cox univariate analysis on all other covariates revealed that common factors (tumour size, perineural invasion, nodal status, differentiation grade, and resection margins) were associated with OS and DSS (Table S3). The Cox multivariable analysis showed NSND patients had similar OS (aHR (95% CI) 1.13, 0.58–2.22, *p* = 0.72) and DSS (aHR (95% CI) 0.92, 0.42–2.02, *p* = 0.84) compared to SD patients when adjusted for confounders (Table 2).

**TABLE 2:** Multivariable Cox regression analysis of patient and tumour characteristics for overall survival (*N* = 45), disease‐specific survival (*N* = 26), second events (*N* = 44), recurrence (*N* = 29) and SPT (*N* = 13).

Variables	OS			DSS			Second events			Recurrence			Second primary tumour		
	HR	95% CI	*p*	HR	95% CI	*p*	HR	95% CI	*p*	HR	95% CI	*p*	HR	95% CI	*p*
Intoxication															
NSND	Ref.			Ref.			Ref.			Ref.			Ref.		
SD	1.13	0.58–2.22	0.72	0.92	0.42–2.02	0.84	0.50	0.26–0.93	**0.03**	0.55	0.25–1.21	0.14	0.25	0.08–0.81	**0.02**
Age, years	1.03	1.00–1.06	**0.02**	1.01	0.98–1.05	0.33	0.98	0.95–1.00	0.07	0.97	0.94–1.00	0.05	0.98	0.93–1.04	0.53
Gender															
Female	Ref.			Ref.			Ref.			Ref.			Ref.		
Male	0.66	0.35–1.24	0.19	0.61	0.29–1.31	0.20	0.85	0.45–1.61	0.62	0.86	0.39–1.87	0.70	1.22	0.37–4.04	0.74
Differentiation															
Well/moderate	NS			NS			NS			Ref.			NS		
Poor										3.35	1.39–8.04	**0.007**			
Tumour size	NS			NS			1.03	1.01–1.05	**< 0.001**	1.05	0.99–1.05	**0.06**	1.05	1.02–1.09	**0.002**
Nodal status															
N0	Ref.			Ref.			Ref.			Ref.			Ref		
N+	3.71	1.87–7.38	**< 0.001**	3.91	1.71–8.91	**0.001**	4.24	2.20–8.17	**< 0.001**	3.91	1.70–8.99	**0.01**	4.59	1.38–15.35	**0.01**
PI															
Absent	Ref.			Ref.			NS			NS			NS		
Present	3.24	1.76–5.99	**< 0.001**	3.10	1.53–6.26	**0.002**									
Margins															
≥ 3 mm	NS			Ref.			NS			NS			NS		
< 3 mm				1.95	0.96–3.96	0.06									

Abbreviations: DSS, disease‐specific survival; NA, not applicable; NS, not significant; OS, overall survival; PI, perineural invasion; SPT, second primary tumour. Bold values signify *p*‐value < 0.05.

### 3.3 Second Events

To identify the differences in second events (recurrence, distant metastasis, SPT) after treatment between NSND and SD patients, Kaplan–Meier analyses were performed. The NSND group had similar risk of a second event in the follow‐up (39.2% vs. 26.3%, log‐rank, *p* = 0.10) (Figure 1D). Kaplan–Meier analysis showed similar risk of recurrence for both groups (21.2% vs. 16.0%, log‐rank *p* = 0.42) (Figure 1C). The NSND group had a significantly higher risk of developing an SPT (23.3% vs. 9.3%, log‐rank *p* = 0.04) (Figure 1E). To ensure the representativeness of this cohort, univariate Cox analysis was performed on all prognostic indicators for second events, recurrences and SPT (Table S3). Univariate Cox analysis showed that NSND patients and SD patients had similar risk of second events (HR (95% CI) 0.61, 0.33–1.11, *p* = 0.11) and recurrences (HR (95% CI) 0.73, 0.34–1.57, *p* = 0.42) (Table S4). The univariate Cox analysis showed a significantly lower hazard ratio for SPT for SD patients (HR (95% CI) 0.34, 0.11–1.01, *p* = 0.05) (Table S2). In order to adjust for possible confounders, we performed multivariable Cox regression analysis (Table 2). This analysis revealed that SD patients had a significantly lower risk of a second event after treatment (aHR (95% CI) 0.50, 0.26–0.93, *p* = 0.03). This is not due to recurrences as the NSND group had a similar risk for recurrence compared to SD patients when adjusted for confounders (aHR (95% CI) 0.55, 0.25–1.21, *p* = 0.14). Because of the increased risk of SPTs in the NSND group, we analysed this outcome in the multivariable analysis. This revealed a lower risk of SPTs for SD patients (aHR (95% CI) 0.25, 0.08–0.81, *p* = 0.02) when adjusted for confounders (Table 2). Using SD as a reference group revealed an adjusted hazard ratio of 3.92 for NSND patients of developing an SPT (aHR (95% CI) 3.92, 1.23–12.48, *p* = 0.02). To assess if deaths or other events were competing event, a sensitivity analysis using multivariable competing risk analysis was performed. This analysis revealed the NSND group had a higher risk of developing an SPT (sHR (95% CI) 2.82, 1.01–7.89, *p* = 0.048) (Table S4).

Differences between included and non‐included patients showed that patients treated with primary (chemo)radiotherapy, palliative or no treatment had multiple comorbidities, older age, advanced tumour stage and had a higher rate of patients with a history of smoking and drinking (Table S5).

## 4 Discussion

This study is, to our knowledge, the first well‐defined prospective cohort of primary OSCC patients treated by surgery with curative intent, designed to compare non‐smoking and non‐drinking (NSND) patients with smoking and/or drinking (SD) patients. Results showed that NSND patients are typically older, more frequently female and more likely to present with OSCC of the tongue, with almost no tumours located on the floor of the mouth. A key finding was that NSND patients exhibited a 3.9‐fold increased risk of developing SPTs during follow‐up compared to SD patients. No significant differences were observed in other clinical or histopathological characteristics and treatments between the groups. Multivariable analysis revealed no difference in locoregional recurrences. Overall survival and disease‐specific survival were similar for NSND patients compared to SD patients in the univariate and multivariable analyses.

OSCC traditionally has been a disease of male smokers and drinkers, which have more than a fivefold increased risk of developing oral cancer (Mello et al. 2019). However, our study showed that NSND patients represent 32.4% of OSCC patients in this cohort. These high rates are similar to other recent studies (Yan et al. 2022). A higher female rate and higher age in the NSND group are in concordance with observations of other studies on smoking and drinking stratification (Adeoye et al. 2021; DeAngelis et al. 2018; Kruse, Bredell, and Gratz 2010; Loeffelbein et al. 2017; van Imhoff et al. 2016; Yan et al. 2022). Higher rates of tongue tumours and lower rates of the floor‐of‐mouth tumours among NSND patients are in line with other reports (Adeoye et al. 2021). We hypothesise that the difference in anatomical locations might be due to the pooling of alcohol and tobacco carcinogens at the floor of the mouth, similar to patterns observed in buccal and gingival cancer among betel quid chewers (Reichart et al. 2008).

Importantly, NSND patients had a higher risk of developing an SPT in the follow‐up. This result was confirmed in the multivariable analysis correcting for known confounders. This finding contrasts with a recent retrospective cohort study that found similar recurrence‐free survival (RFS) between the two groups (Yan et al. 2022). A similar study showed no differences in SPTs (Koo et al. 2013). Conflicting results in outcome can be explained by the different study designs. A recent review showed 17 out of 20 studies on this topic were retrospective cohorts (Adeoye et al. 2021). Furthermore, NSND studies often include a mix of locations of SCC in the head and neck region (Brennan et al. 2017; Farshadpour et al. 2007; Moyses et al. 2013; Wiseman et al. 2003) or other histology subtypes (Bao et al. 2020). Other outcomes were consistent with previous research, as this study showed no significant disparities in OS and DSS between SD and NSND patients (Kruse, Bredell, and Gratz 2010; Moyses et al. 2013). The current study's findings highlight comparable 5‐year survival rates for OS and DSS among the entire study population, especially 66.6% for OS and 83.2% for DSS, which align closely with rates reported in other large cohort studies (van Dijk et al. 2016; Weckx et al. 2020; Zanoni et al. 2019).

In the absence of etiological factors, we must consider other possible biological factors as driver of tumourigenesis. Oral premalignant disorders (OPMDs), such as leukoplakia, are known to eventually develop oral cancer and have a more aggressive natural history in never smokers (Warnakulasuriya et al. 2021). However, data on history of OPMDs were not collected in this study, and their effect could not be determined. We hypothesise that this could be a factor in the development of SPT in NSND patients. The concept of ‘field cancerization’ is often linked to the development of SPTs; however, this concept typically pertains to carcinogenic damage in the SD group. Our results demonstrated the contrary; therefore, this remains unresolved. Recent research has advanced our understanding of NSND patients' molecular profile, revealing significant mutations such as CDKN2A, EGFR amplifications and BRCA2 deletions. These findings suggest potential biomarkers that could explain the development of oral tumours in NSND patients (Koo et al. 2021). Furthermore, a comprehensive analysis of the tumour microenvironment showed overexpressing of *IDO1* and *PD‐L1*, enrichment of *IFN‐γ* and *PD1* pathways, and a higher intratumour T‐cell infiltrate in NSND compared to SD OSCC patients (Foy et al. 2017). Despite these advancements, further understanding of the molecular distinctions between these groups and their prognostic significance in the development of SPTs is needed.

The strengths of our study lie in its consistent prospective data collection and treatment protocols, enhancing the reliability of the findings. Stage information was present, but raw histopathological data on patient and tumour characteristics were used. This enabled adjustment for known prognostic confounders in the multivariable analysis. The prognostic factors that we identified, such as older age, increasing tumour size, depth of invasion, poor differentiation grade, presence of perineural invasion, positive nodal status, and presence of extranodal extension, are already well established in current literature and show that this is a representative OSCC patient cohort, that is, treated by surgery with curative intent. However, a limitation of this study is that the inclusion criteria may restrict the generalisability of our findings to all OSCC patients, particularly those treated with chemoradiotherapy or managed with palliative care. Another limitation is the absence of data on oral history, such as OPMDs, which could elucidate the development of SPTs in the follow‐up period of NSND patients.

## 5 Conclusion

We demonstrated that NSND is a subgroup of OSCC patients with differences in patient and tumour characteristics. With an ageing population and declining usage of tobacco and alcohol consumption in the Netherlands, NSND becomes an increasingly important subgroup to observe and study. Our observations warrant a closer examination of this subgroup more allowing earlier detection of second primary tumours. Lastly, the lack of the traditional risk factors smoking and alcohol use leaves the question why these patients are developing these tumours and why the characteristics of the patient and tumour are different. Lack of a clear explanation for the differences in prognosis necessitates further research. Future NSND studies should explore the potential role of oral premalignant disorders and conduct tumour mutational profiling using, for example, as next‐generation sequencing.

## Author Contributions

**Pepijn J. P. van der Aa:** conceptualization, investigation, writing – original draft, methodology, validation, visualization, formal analysis, project administration. **Max J. H. Witjes:** writing – review and editing, supervision. **Bert van der Vegt:** writing – review and editing, supervision. **Ed Schuuring:** writing – review and editing, supervision. **Koos Boeve:** writing – review and editing. **Grigory Sidorenkov:** formal analysis, methodology. **Geertruida H. de Bock:** formal analysis, supervision, conceptualization, methodology, writing – review and editing, validation, resources. **Sebastiaan A. H. J. de Visscher:** conceptualization, methodology, writing – review and editing, supervision, validation, project administration, resources.

## Conflicts of Interest

The authors declare no conflicts of interest.

## Supporting information


Data S1.

## Data Availability

The data that support the findings of this study are available on request from the corresponding author. The data are not publicly available due to privacy or ethical restrictions.

## References

[odi15235-bib-0001] Adeoye, J. , J. Y. Tan , C. M. Ip , S. W. Choi , and P. Thomson . 2021. “Fact or Fiction?: Oral Cavity Cancer in Nonsmoking, Nonalcohol Drinking Patients as a Distinct Entity—Scoping Review.” Head and Neck 43, no. 11: 3662–3680. 34313348 10.1002/hed.26824

[odi15235-bib-0002] Amin, M. B. M. D. , S. B. Edge , and F. L. Greene . 2017. AJCC Cancer Staging Manual (Eight). New York, NY: Springer.

[odi15235-bib-0003] Bao, X. , F. Liu , Q. Chen , et al. 2020. “Propensity Score Analysis Exploring the Impact of Smoking and Drinking on the Prognosis of Patients With Oral Cancer.” Head & Neck 42, no. 8: 1837–1847. 32031313 10.1002/hed.26099

[odi15235-bib-0004] Braakhuis, B. J. , C. R. Leemans , and O. Visser . 2014. “Incidence and Survival Trends of Head and Neck Squamous Cell Carcinoma in The Netherlands Between 1989 and 2011.” Oral Oncology 50, no. 7: 670–675. 24735546 10.1016/j.oraloncology.2014.03.008

[odi15235-bib-0005] Braakhuis, B. J. , M. P. Tabor , C. R. Leemans , I. van der Waal , G. B. Snow , and R. H. Brakenhoff . 2002. “Second Primary Tumors and Field Cancerization in Oral and Oropharyngeal Cancer: Molecular Techniques Provide New Insights and Definitions.” Head & Neck 24, no. 2: 198–206. 11891950 10.1002/hed.10042

[odi15235-bib-0006] Brennan, K. , J. L. Koenig , A. J. Gentles , J. B. Sunwoo , and O. Gevaert . 2017. “Identification of an Atypical Etiological Head and Neck Squamous Carcinoma Subtype Featuring the CpG Island Methylator Phenotype.” eBioMedicine 17: 223–236. 28314692 10.1016/j.ebiom.2017.02.025 PMC5360591

[odi15235-bib-0007] Bruggink, J. W. 1991. “Ontwikkelingen in Het Aandeel Rokers in Nederland Sinds 1989.” TSG 4: 234–240.

[odi15235-bib-0008] Charlson, M. , T. P. Szatrowski , J. Peterson , and J. Gold . 1994. “Validation of a Combined Comorbidity Index.” Journal of Clinical Epidemiology 47, no. 11: 1245–1251. 7722560 10.1016/0895-4356(94)90129-5

[odi15235-bib-0009] Chegini, S. , C. Schilling , E. S. Walgama , et al. 2021. “Neck Failure Following Pathologically Node‐Negative Neck Dissection (pN0) in Oral Squamous Cell Carcinoma: A Systematic Review and Meta‐Analysis.” British Journal of Oral & Maxillofacial Surgery 59, no. 10: 1157–1165. 34281738 10.1016/j.bjoms.2021.04.002

[odi15235-bib-0010] DeAngelis, A. , O. Breik , K. Koo , et al. 2018. “Non‐smoking, Non‐drinking Elderly Females, a 5 Year Follow‐Up of a Clinically Distinct Cohort of Oral Squamous Cell Carcinoma Patients.” Oral Oncology 86: 113–120. 30409291 10.1016/j.oraloncology.2018.09.004

[odi15235-bib-0011] Farshadpour, F. , G. J. Hordijk , R. Koole , and P. J. Slootweg . 2007. “Non‐smoking and Non‐drinking Patients With Head and Neck Squamous Cell Carcinoma: A Distinct Population.” Oral Diseases 13, no. 2: 239–243. 17305629 10.1111/j.1601-0825.2006.01274.x

[odi15235-bib-0012] Fine, J. P. , and R. J. Gray . 1999. “A Proportional Hazards Model for the Subdistribution of a Competing Risk.” Journal of the American Statistical Association 94, no. 446: 496–509.

[odi15235-bib-0013] Foy, J. P. , C. Bertolus , M. C. Michallet , et al. 2017. “The Immune Microenvironment of HPV‐Negative Oral Squamous Cell Carcinoma From Never‐Smokers and Never‐Drinkers Patients Suggests Higher Clinical Benefit of IDO1 and PD1/PD‐L1 Blockade.” Annals of Oncology 28, no. 8: 1934–1941. 28460011 10.1093/annonc/mdx210

[odi15235-bib-0014] IARC Working Group on the Evaluation of Carcinogenic Risks to Humans . 2004. “Tobacco Smoke and Involuntary Smoking.” IARC Monographs on the Evaluation of Carcinogenic Risks to Humans 83: 1–1438. 15285078 PMC4781536

[odi15235-bib-0015] IARC Working Group on the Evaluation of Carcinogenic Risks to Humans . 2010. “Alcohol Consumption and Ethyl Carbamate.” IARC Monographs on the Evaluation of Carcinogenic Risks to Humans 96: 3–1383. 21735939 PMC4781168

[odi15235-bib-0016] Koo, K. , R. Barrowman , M. McCullough , T. Iseli , and D. Wiesenfeld . 2013. “Non‐Smoking Non‐Drinking Elderly Females: A Clinically Distinct Subgroup of Oral Squamous Cell Carcinoma Patients.” International Journal of Oral and Maxillofacial Surgery 42, no. 8: 929–933. 23702369 10.1016/j.ijom.2013.04.010

[odi15235-bib-0017] Koo, K. , D. Mouradov , C. M. Angel , et al. 2021. “Genomic Signature of Oral Squamous Cell Carcinomas From Non‐Smoking Non‐Drinking Patients.” Cancers 13, no. 5: 1029. 33804510 10.3390/cancers13051029 PMC7957667

[odi15235-bib-0018] Kruse, A. L. , M. Bredell , and K. W. Gratz . 2010. “Oral Squamous Cell Carcinoma in Non‐Smoking and Non‐Drinking Patients.” Head & Neck Oncology 2: 24. 20920351 10.1186/1758-3284-2-24 PMC2958869

[odi15235-bib-0019] Loeffelbein, D. , L. M. Ritschl , F. D. Gull , M. Roth , K. D. Wolff , and T. Mucke . 2017. “Influence of Possible Predictor Variables on the Outcome of Primary Oral Squamous Cell Carcinoma: A Retrospective Study of 392 Consecutive Cases at a Single Centre.” International Journal of Oral and Maxillofacial Surgery 46, no. 4: 413–421. 28007325 10.1016/j.ijom.2016.11.014

[odi15235-bib-0020] Maasland, D. H. , P. A. van den Brandt , B. Kremer , R. A. Goldbohm , and L. J. Schouten . 2014. “Alcohol Consumption, Cigarette Smoking and the Risk of Subtypes of Head‐Neck Cancer: Results From The Netherlands Cohort Study.” BMC Cancer 14: 187. 24629046 10.1186/1471-2407-14-187 PMC4004328

[odi15235-bib-0021] Mello, F. W. , G. Melo , J. J. Pasetto , C. A. B. Silva , S. Warnakulasuriya , and E. R. C. Rivero . 2019. “The Synergistic Effect of Tobacco and Alcohol Consumption on Oral Squamous Cell Carcinoma: A Systematic Review and Meta‐Analysis.” Clinical Oral Investigations 23, no. 7: 2849–2859. 31111280 10.1007/s00784-019-02958-1

[odi15235-bib-0022] Moyses, R. A. , R. V. M. López , P. M. Cury , et al. 2013. “Significant Differences in Demographic, Clinical, and Pathological Features in Relation to Smoking and Alcohol Consumption Among 1,633 Head and Neck Cancer Patients.” Clinics 68, no. 6: 738–744. 23778492 10.6061/clinics/2013(06)03 PMC3674275

[odi15235-bib-0023] Nederlandse Werkgroep Hoofd‐halstumoren . 2004. “Richtlijn Mondholte–en Orofarynxcarcinoom. Alphen aan den Rijn.”

[odi15235-bib-0024] Netherlands Cancer Registry (NCR) . 2024. “Netherlands Comprehensive Cancer Organisation (IKNL).” www.iknl.nl/en/ncr/ncr‐data‐figures.

[odi15235-bib-0036] Reichart, P. A. , and X. H. Nguyen . 2008. “Betel quid chewing, oral cancer and other oral mucosal diseases in Vietnam: a review.” Journal of Oral Pathology & Medicine 37: 511–514. 18624933 10.1111/j.1600-0714.2008.00669.x

[odi15235-bib-0025] Schuurmans, H. , N. Steverink , S. Lindenberg , N. Frieswijk , and J. P. J. Slaets . 2004. “Old or Frail: What Tells Us More?” Journals of Gerontology Series A: Biological Sciences and Medical Sciences 59, no. 9: 962–965. 10.1093/gerona/59.9.m962 15472162

[odi15235-bib-0026] Sidorenkov, G. , J. Nagel , C. Meijer , et al. 2019. “The OncoLifeS Data‐Biobank for Oncology: A Comprehensive Repository of Clinical Data, Biological Samples, and the patient's Perspective.” Journal of Translational Medicine 17, no. 1: 374. 31727094 10.1186/s12967-019-2122-x PMC6857242

[odi15235-bib-0027] van Dijk, B. A. C. , M. T. Brands , S. M. E. Geurts , M. A. W. Merkx , and J. L. N. Roodenburg . 2016. “Trends in Oral Cavity Cancer Incidence, Mortality, Survival and Treatment in The Netherlands.” International Journal of Cancer 139, no. 3: 574–583. 27038013 10.1002/ijc.30107

[odi15235-bib-0028] van Imhoff, L. C. , G. G. Kranenburg , S. Macco , et al. 2016. “Prognostic Value of Continued Smoking on Survival and Recurrence Rates in Patients With Head and Neck Cancer: A Systematic Review.” Head & Neck 38, no. Suppl 1: E2214–E2220. 25900211 10.1002/hed.24082

[odi15235-bib-0029] van Laar, M. W. , R. J. J. van Beek , E. M. T. Beenakkers , et al. 2021. “Nationale Drugmonitor Kerncijfers en Ontwikkelingen 2021, Utrecht Trimbos‐Instituut.”

[odi15235-bib-0030] Warnakulasuriya, S. , O. Kujan , J. M. Aguirre‐Urizar , et al. 2021. “Oral Potentially Malignant Disorders: A Consensus Report From an International Seminar on Nomenclature and Classification, Convened by the WHO Collaborating Centre for Oral Cancer.” Oral Diseases 27, no. 8: 1862–1880. 33128420 10.1111/odi.13704

[odi15235-bib-0032] Weckx, A. , K. J. Grochau , A. Grandoch , T. Backhaus , J. E. Zoller , and M. Kreppel . 2020. “Survival Outcomes After Surgical Treatment of Oral Squamous Cell Carcinoma.” Oral Diseases 26, no. 7: 1432–1439. 32428375 10.1111/odi.13422

[odi15235-bib-0033] Wiseman, S. M. , H. Swede , D. L. Stoler , et al. 2003. “Squamous Cell Carcinoma of the Head and Neck in Nonsmokers and Nondrinkers: An Analysis of Clinicopathologic Characteristics and Treatment Outcomes.” Annals of Surgical Oncology 10, no. 5: 551–557. 12794022 10.1245/aso.2003.09.010

[odi15235-bib-0034] Yan, E. Z. , B. M. Wahle , E. R. Nakken , et al. 2022. “No Survival Benefit in Never‐Smoker Never‐Drinker Patients With Oral Cavity Cancer.” Head and Neck 45: 1–11. 10.1002/hed.27266 PMC9898183 36524736

[odi15235-bib-0035] Zanoni, D. K. , P. H. Montero , J. C. Migliacci , et al. 2019. “Survival Outcomes After Treatment of Cancer of the Oral Cavity (1985–2015).” Oral Oncology 90: 115–121. 30846169 10.1016/j.oraloncology.2019.02.001 PMC6417804

