# Supplementary material for: Non‐smoking and Non‐drinking Oral Cancer Patients Are at Higher Risk of Second Primary Tumours
Source: Oral Dis. 2024 Dec 30;31(7):2129–38. doi: 10.1111/odi.15235 (PMC12319359; doi:10.1111/odi.15235)
Supplement: Supplementary file 1 — Data S1. [file ODI-31-2129-s001.zip › odi15235-sup-0002-Supinfo2.docx]

Supplementary Figure 1. Flowchart diagram showing the in- and exclusion of patients.

**
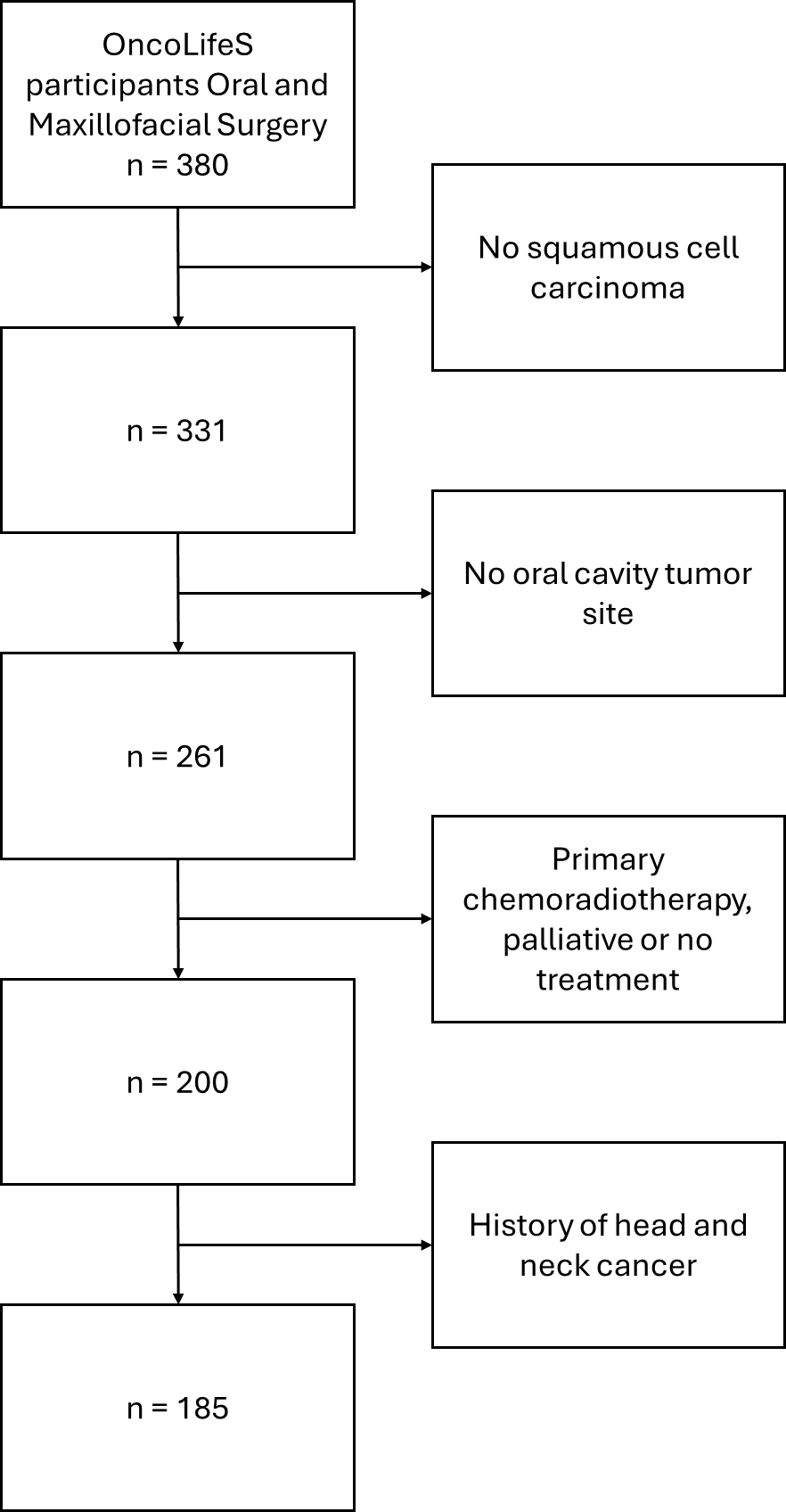
**

Supplementary Table 1. Description of follow-up and outcomes stratified for NSND (N=60) and SD (N=125), (N is observed count of events, percentages is estimated risk of event or survival).

|  | Total | | NSND | | SD | | p-value |
| --- | --- | --- | --- | --- | --- | --- | --- |
|  | N | % | N | % | N | % |  |
| Follow-up time  (median ± IQR) | 36.0 | 14.0-62.5 | 34.5 | 14.0-56.7 | 37.0 | 14.5- 65.0 | 0.30 |
| Local-regional recurrence  1-year  2-year | 23  28 | 13.7%  17.7% | 10  11 | 17.2%  21.2% | 13  17 | 11.5%  16.0% | 0.42 |
| Second primary tumour  1-year  2-year  5-year | 1  3  13 | 0.7%  2.3%  13.5% | 0  2  7 | 5.4%  23.3% | 1  1  6 | 1.0%  1.0%  9.3% | 0.04 |
| Overall survival  1-year  2-year  5-year | 17  30  45 | 80.4%  81.7%  66.6% | 5  12  16 | 81.4%  76.4%  61.9% | 12  18  29 | 89.9%  84.0%  68.6% | 0.49 |
| Disease specific survival  1-year  2-year  5-year | 13  23  26 | 92.7%  85.9%  83.2% | 4  9  10 | 93.1%  82.5%  79.5% | 9  14  16 | 82.4%  87.4%  84.9% | 0.47 |

Supplementary Table 2. Univariate Cox regression analysis for survival data; OS (N = 45) and DSS (N = 26)

|  | OS | | | DSS | | |
| --- | --- | --- | --- | --- | --- | --- |
| **Variables** | **HR** | **95% CI** | **p** | **HR** | **95% CI** | **p** |
| Age, years | 1.04 | 1.01-1.07 | 0.01 | 1.01 | 0.97-1.04 | 0.62 |
| Gender  Female  Male | Ref.  1.58 | 0.88-2.84 | 0.13 | Ref.  1.68 | 0.77-3.66 | 0.19 |
| BMI  Normal  Under–or overweight | Ref.  0.80 | 0.44-1.44 | 0.45 | Ref.  0.60 | 0.28-1.30 | 0.19 |
| CCI  <5  ≥5 | Ref.  3.65 | 1.76-7.59 | **<0.001** | Ref.  1.92 | 0.83-4.42 | 0.12 |
| GFI  <4  ≥4 | Ref.  1.10 | 0.55-2.21 | 0.78 | Ref.  1.33 | 0.57-3.09 | 0.50 |
| Intoxication  NSND  SD | Ref.  0.81 | 0.44-1.49 | 0.49 | Ref.  0.74 | 0.34-1.64 | 0.46 |
| Tumour site  Tongue  Gum  Floor of mouth  Palate  Buccal mucosa | Ref.  1.58  1.32  ID  1.59 | 0.76-3.31  0.58-3.01  0.64-3.97 | 0.74  0.22  0.50  0.32 | Ref.  1.48  0.76  ID  1.22 | 0.59-3.71  0.22-2.67  0.35-4.29 | 0.88  0.40  0.67  0.75 |
| Differentiation grade  Well  Moderate  Poor | Ref.  5.45  11.77 | 1.68-17.7  2.94-47.15 | **<0.01**  **<0.01**  **<0.001** | Ref.  9.55  24.43 | 1.28-71.02  2.73-218.66 | **0.04**  **0.03**  **0.00** |
| Tumour size, mm | 1.03 | 1.01-1.04 | **<0.01** | 1.03 | 1.00-1.05 | **0.03** |
| Tumour size  <20  20-40  ≥40 | Ref.  1.72  2.21 | 0.91-3.24  0.93-5.26 | 0.11  0.10  0.07 | Ref.  1.76  2.08 | 0.76-4.05  0.66-6.53 | 0.30  0.19  0.21 |
| Nodal status  N0  N+ | Ref.  5.58 | 2.82-11.04 | **<0.001** | Ref.  7.09 | 2.67-18.83 | **<0.001** |
| DOI, mm | 1.07 | 1.03-1.10 | **<0.001** | 1.09 | 1.04-1.13 | **<0.001** |
| DOI  <5 mm  5 - 10 mm  10 - 20 mm  ≥20 mm | Ref.  2.27  4.44  4.77 | 0.98-5.26  1.96-10.07  1.70-13.42 | **<0.01**  **0.05**  **<0.001**  **<0.01** | Ref.  2.56  6.07  9.21 | 0.75-8.75  1.90-19.37  2.47-34.30 | **0.01**  0.13  **<0.001**  **<0.001** |
| Resection margins  >5 mm  4-5 mm  3-4 mm  2-3 mm  1-2 mm  <1 mm | Ref.  0.56  0.90  1.50  1.55  2.56 | 0.13-2.40  0.30-2.64  0.51-4.45  0.58-4.15  1.22-5.39 | 0.13  0.43  0.84  0.46  0.38  **0.01** | Ref.  0.65  1.11  2.27  2.16  4.74 | 0.08-5.22  0.24-5.23  0.60-8.57  0.57-8.13  1.83-12.30 | **0.03**  0.69  0.89  0.23  0.26  **<0.01** |
| Marge groups  ≥3  1-3  <1 | Ref.  1.65  2.77 | 0.77-3.55  1.36-5.63 | **0.02**  0.20  **<0.01** | Ref.  2.28  4.89 | 0.84-6.16  2.02-11.80 | **<0.01**  0.10  **<0.001** |
| PI  Absent  Present | Ref.  4.45 | 2.48-8.00 | **<0.001** | Ref.  5.58 | 2.56-12.18 | **<0.001** |
| LVI  Absent  Present | Ref.  1.86 | 0.90-3.87 | 0.10 | Ref.  2.34 | 0.94-5.83 | 0.07 |
| ENE  Absent  Present | Ref.  5.15 | 2.75-9.63 | **<0.001** | Ref.  6.63 | 3.04-14.46 | **<0.001** |

Abbreviations: **BMI**, Body Mass Index kg/m2; **CCI**, Charlson Comorbidity Index; **GFI**, Groningen Frailty Indicator; **DOI**, depth of invasion; **PI**, perineural invasion; **LVI**, Lymphovascular invasion; **ENE**, extra nodal extension.

Supplementary Table 3. Univariate Cox regression analysis for recurrence (N = 29), second event (N = 44) and second primary tumour (N = 13).

|  | Recurrence | | | Second event | | | Second primary tumour | | |
| --- | --- | --- | --- | --- | --- | --- | --- | --- | --- |
| Variables | **HR** | **95% CI** | **p** | **HR** | **95% CI** | **p** | **HR** | **95% CI** | **p** |
| Age, years | 0.98 | 0.95-1.01 | 0.27 | 0.99 | 0.96-1.01 | 0.40 | 1.02 | 0.97-1.08 | 0.36 |
| Gender  Female  Male | Ref.  2.21 | 0.82-5.98 | 0.12 | Ref.  1.57 | 0.86-2.87 | 0.14 | Ref.  1.12 | 0.38-3.34 | 0.84 |
| BMI  18.5-24.99 kg/m2  Other | Ref.  0.52 | 0.25-1.10 | 0.09 | Ref.  0.63 | 0.35-1.15 | 0.13 | Ref.  1.54 | 0.47-5.02 | 0.47 |
| CCI  <5  ≥5 | Ref.  0.92 | 0.44-1.94 | 0.83 | Ref.  0.93 | 0.51-1.69 | 0.81 | Ref.  0.97 | 0.33-2.90 | 0.96 |
| GFI  <4  ≥4 | Ref.  0.86 | 0.35-2.12 | 0.74 | Ref.  0.63 | 0.28-1.43 | 0.56 | Ref.  0.26 | 0.03-2.02 | 0.20 |
| Intoxication  NSND  SD | Ref.  0.73 | 0.34-1.57 | 0.42 | Ref.  0.61 | 0.33-1.11 | 0.11 | Ref.  0.34 | 0.11-1.01 | 0.05 |
| Tumour site  Tongue  Gum  Floor of mouth  Palate  Buccal mucosa | Ref.  0.70  0.37  ID  0.80 | 0.26-1.88  0.09-1.59  0.24-2.73 | 0.72  0.47  0.18  0.73 | Ref.  1.08  1.04  ID  1.16 | 0.50-2.35  0.45-2.45  0.44-3.01 | 1.00  0.84  0.92  0.76 | Ref.  2.78  2.43  ID  2.62 | 0.69-11.1  0.54-10.9  0.48-14.3 | 0.63  0.15  0.24  0.27 |
| Differentiation grade  Well  Moderate  Poor | Ref.  2.66  10.52 | 0.78-9.08  2.51-44.1 | **0.01**  0.12  **0.001** | Ref.  2.31  5.82 | 0.96-5.57  1.77-19.1  1.72-11.5 | **0.03**  0.06  **0.001** | Ref.  1.58  ID | 0.43-5.74 | 0.92  0.49 |
| Tumour size, mm | 1.02 | 1.00-1.05 | 0.07 | 1.04 | 1.02-1.06 | **0.001** | 1.04 | 1.01-1.08 | **0.01** |
| Tumour size  <20  20-40  ≥40 | Ref.  0.98  2.62 | 0.41-2.33  1.01-6.84 | 0.11  0.96  0.05 | Ref.  1.71  2.82 | 0.88-3.32  1.22-6.51 | **0.04**  0.11  **0.01** | Ref.  3.14  3.88 | 0.92-10.7  0.71-21.2 | 0.14  0.07  0.12 |
| Nodal status  N0  N+ | Ref.  4.20 | 1.85-9.54 | **<0.001** | Ref.  4.33 | 2.26-8.32 | **<0.001** | Ref.  3.54 | 1.16-10.8 | **0.03** |
| DOI, mm | 1.05 | 1.01-1.10 | **0.02** | 1.07 | 1.04-1.11 | **<0.001** | 1.09 | 1.03-1.15 | **0.001** |
| DOI  <5 mm  5 - 10 mm  10 - 20 mm  ≥20 mm | Ref.  1.47  4.55  2.05 | 0.52-4.20  1.79-11.6  0.43-9.9 | **0.02**  0.47  **0.001**  0.37 | Ref.  1.66  3.99  5.16 | 0.70-3.91  1.79-8.90  1.96-13.6 | **0.001**  0.25  **<0.001**  **<0.001** | Ref.  1.99  1.98  9.60 | 0.44-8.88  0.33-11.9  2.15-42.9 | **0.04**  0.37  0.45  **<0.001** |
| Resection margins  >5 mm  4-5 mm  3-4 mm  2-3 mm  1-2 mm  <1 mm | Ref.  0.89  0.82  1.88  1.76  2.79 | 0.20-4.04  0.18-3.72  0.52-6.74  0.49-6.32  1.08-7.21 | 0.31  0.89  0.80  0.33  0.38  **0.03** | Ref.  0.96  0.82  2.57  1.90  2.80 | 0.28-3.31  0.21-2.83  1.00-6.60  0.70-5.19  1.27-6.18 | 0.07  0.95  0.76  **0.05**  0.21  **0.01** | Ref.  1.16  0..91  3.94  1.36  2.83 | 0.13-9.9  0.11-7.78  0.76-20.5  0.16-11.7  0.68-11.9 | 0.53  0.89  0.93  0.10  0.78  0.15 |
| Marge groups  ≥3  1-3  <1 | Ref.  1.90  2.92 | 0.74-4.89  1.19-7.16 | 0.05  0.18  **0.02** | Ref.  2.29  2.90 | 1.11-4.74  1.37-6.13 | **<0.01**  **0.02**  **<0.001** | Ref.  2.40  2.82 | 0.62-9.32  0.73-10.9 | 0.22  0.21  0.13 |
| PI  Absent  Present | Ref.  3.90 | 1.85-8.21 | **<0.001** | Ref.  3.16 | 1.72-5.80 | **<0.001** | Ref.  3.22 | 1.05-9.86 | **0.04** |
| LVI  Absent  Present | Ref.  2.32 | 0.94-5.72 | 0.07 | Ref.  2.53 | 1.25-5.14 | **0.01** | Ref.  3.68 | 1.13-11.96 | **0.03** |
| ENE  Absent  Present | Ref.  3.42 | 1.45-8.06 | **<0.01** | Ref.  4.60 | 2.34-9.06 | **<0.001** | Ref.  8.83 | 2.61-29.8 | **<0.001** |

Abbreviations: **BMI**, Body Mass Index kg/m2; **CCI**, Charlson Comorbidity Index; **GFI**, Groningen Frailty Indicator; **DOI**, depth of invasion; **PI**, perineural invasion; **LVI**, Lymphovascular invasion; **ENE**, extra nodal extension.

Supplementary Table 4. Multivariable Competing Risk Analysis for second events (N = 44), recurrence (N = 29) and SPT (N =13).

|  | Second events | | | Recurrence | | | Second primary tumour | | |
| --- | --- | --- | --- | --- | --- | --- | --- | --- | --- |
| Variables | **HR** | **95% CI** | **p** | **HR** | **95% CI** | **p** | **HR** | **95% CI** | **p** |
| Intoxication   - SD - NSND | Ref.  1.71 | 0.94-3.12 | 0.08 | Ref.  1.42 | 0.67-3.01 | 0.36 | Ref.  2.82 | 1.01 -7.89 | **0.048** |
| Differentiation grade   - Well/moderate - Poor | NS |  |  | Ref.  2.65 | 1.17 -5.98 | **0.02** | NS |  |  |
| Tumour size | 1.03 | 1.01-1.05 | **0.009** | 1.05 | 1.02-1.09 | 0.18 | 1.04 | 1.02-1.69 | **0.002** |
| Nodal status   - N0 - N+ | Ref.  4.24 | 2.20-8.17 | **<0.001** | Ref.  2.93 | 1.23-6.96 | **0.002** | Ref  2.28 | 0.79-6.52 | 0.13 |
